# Predictable enriched environment prevents development of hyper-emotionality in the VPA rat model of autism

**DOI:** 10.3389/fnins.2015.00127

**Published:** 2015-06-02

**Authors:** Mônica R. Favre, Deborah La Mendola, Julie Meystre, Dimitri Christodoulou, Melissa J. Cochrane, Henry Markram, Kamila Markram

**Affiliations:** Laboratory of Neural Microcircuits, Brain Mind Institute, École Polytechnique Fédérale de LausanneLausanne, Switzerland

**Keywords:** autism, valproic acid, predictability, environmental enrichment, emotion, individual differences, protein expression, rat

## Abstract

Understanding the effects of environmental stimulation in autism can improve therapeutic interventions against debilitating sensory overload, social withdrawal, fear and anxiety. Here, we evaluate the role of environmental predictability on behavior and protein expression, and inter-individual differences, in the valproic acid (VPA) model of autism. Male rats embryonically exposed (E11.5) either to VPA, a known autism risk factor in humans, or to saline, were housed from weaning into adulthood in a standard laboratory environment, an unpredictably enriched environment, or a predictably enriched environment. Animals were tested for sociability, nociception, stereotypy, fear conditioning and anxiety, and for tissue content of glutamate signaling proteins in the primary somatosensory cortex, hippocampus and amygdala, and of corticosterone in plasma, amygdala and hippocampus. Standard group analyses on separate measures were complemented with a composite emotionality score, using Cronbach's Alpha analysis, and with multivariate profiling of individual animals, using Hierarchical Cluster Analysis. We found that predictable environmental enrichment prevented the development of hyper-emotionality in the VPA-exposed group, while unpredictable enrichment did not. Individual variation in the severity of the autistic-like symptoms (fear, anxiety, social withdrawal and sensory abnormalities) correlated with neurochemical profiles, and predicted their responsiveness to predictability in the environment. In controls, the association between socio-affective behaviors, neurochemical profiles and environmental predictability was negligible. This study suggests that rearing in a predictable environment prevents the development of hyper-emotional features in animals exposed to an autism risk factor, and demonstrates that unpredictable environments can lead to negative outcomes, even in the presence of environmental enrichment.

## Introduction

Autism is a pervasive neurodevelopmental disorder, diagnosed behaviorally upon early childhood presentation of social impairment, abnormal communication, and inflexible behaviors. Symptoms lie on a continuum of severity (Autism Spectrum Disorder, DSM-5; American Psychiatric Association, 2013), and are accompanied by a variety of other abnormalities. These include disabling anxiety, phobias, self-injury and aggressiveness, seizures and sleep disturbances, and exceptional abilities in attention, learning and memory (Mottron et al., 2006; Argyropoulos et al., 2013). There is currently no consensus on the underlying neuropathology (Moldin and Rubenstein, 2006; Betancur, 2011; Zahir and Brown, 2011; Parellada et al., 2014). In consequence, there are multiple treatment options, significant variability in individual response to treatment, and no predictors of the effectiveness of specific treatments for specific individuals (Rossignol, 2009; Bent and Hendren, 2010; Vivanti et al., 2014).

A strong preference for behavioral and environmental consistency is a distinctive characteristic of autistic children. Therapeutic approaches with documented effectiveness are mainly based on environmental modification and intensive behavioral training (Gresham et al., 1999; Moldin and Rubenstein, 2006; Bent and Hendren, 2010; Leblanc and Gillis, 2012; Vivanti et al., 2014), where predictability features, such as structure and routine, have been shown to reduce anxiety and problem behaviors, to increase children's positive interactions with the physical and social surrounding (e.g., Flannery and Horner, 1994), and to improve their ability to function in everyday life (Ferrara and Hill, 1980; Kootz et al., 1982; Sterling-Turner and Jordan, 2007; White et al., 2009; Chamberlain et al., 2013). In parallel, an increasing body of evidence suggests that the autistic brain processes surprising events and novel sensory stimuli in an unusual way (Markram and Markram, 2010; Gomot and Wicker, 2012; Zikopoulos and Barbas, 2013; Sinha et al., 2014). It has been proposed that the autism spectrum results from hyper-connected and hyper-plastic neural microcircuits very early in neural development, leading to amplified sensory processing, memory formation and hyper-emotionality. These unusual features of the autistic brain would render the world overly intense, and potentially aversive (Markram et al., 2007; Markram and Markram, 2010). If this is true, exposure to unpredictable environments and over-stimulation could, in fact, accelerate the development of autistic symptoms. However, to this date most research on effective treatment (e.g., Woo and Leon, 2013) and the underlying neurobiology view the autistic child and brain as hypo-functional. Hence, studies have focused on environmental enrichment and stimulation, but have not made the distinction between predictable and unpredictable stimulation (Moldin and Rubenstein, 2006; Leblanc and Gillis, 2012). Studying these issues could provide valuable insights for early diagnosis and individualized therapy.

In this study, therefore, we investigated the impact of rearing environments, with different degrees of enrichment and predictability, on the development of autistic-like behavior, the inter-individual differences, and the associated neurobiology, in an animal model of autism. We used the extensively validated prenatal valproic acid (VPA) rodent model of autism (Chapman and Cutler, 1984; Clayton-Smith and Donnai, 1995; Rodier et al., 1996; Arndt et al., 2005; Kini, 2006; Wagner et al., 2006; Markram et al., 2007; Roullet and Crawley, 2011; Chomiak and Hu, 2012; Argyropoulos et al., 2013; Christensen et al., 2013; Favre et al., 2013; Roullet et al., 2013). VPA has epigenetic effects though histone deacetylase inhibition, influencing developmental cascades and multiple neural functions (Cotariu et al., 1990; Contestabile and Sintoni, 2013; Graff and Tsai, 2013). Thus, this toxin insult-based model is complementary to other well-established gene-based models, given that an environmentally-triggered epigenetic event, such as VPA exposure, can act synergistically with inherited polygenetic factors, and lower the biological threshold for the manifestation of autism. Previous studies have shown that VPA-exposed rats exhibit neuronal hyper-reactivity and hyper-plasticity in the primary somatosensory cortex, medial prefrontal cortex and in the amygdala (Rinaldi et al., 2007, 2008b; Silva et al., 2009; Sui and Chen, 2012; Kim et al., 2013; Martin and Manzoni, 2014). Together with human observations indicating atypical synchronization and connectivity patterns in autism (Markram and Markram, 2010; Courchesne et al., 2011; Gomot and Wicker, 2012; Zikopoulos and Barbas, 2013), these studies raise the possibility that autistic brains are particularly sensitive to environmental stimulation. In 2006, Schneider and colleagues demonstrated that enrichment could reverse autistic-like social deficits, stereotypy and anxiety in VPA exposed rats (Schneider et al., 2006). However, they did not address if predictability was the essential feature in the enriched environment. We further noted that many previous studies on the VPA model did not examine inter-individual variations in responses and their neurobiological correlates, which may be necessary for clinical translation (Bent and Hendren, 2010). Taking these indications together, we hypothesized that enriched but predictable environments would prevent hyper-emotional outcomes, while enriched environments that were unpredictable or standard non-enriched environments would lead to abnormal behaviors; we would also expect that a range of individual responses could be associated with neurobiological differences in sensory or cognitive-affective systems.

To address these questions, we exposed male rats prenatally (E11.5) either to a single dose of saline (SAL) or to VPA (i.p. 500 mg/kg) and housed them in one of three environments defined by *standard* laboratory conditions (ST), *unpredictably enriching* conditions (UE), or *predictably enriching* conditions (PE). First, we studied the effects of prenatal exposure and postnatal home environment on separate behavioral measures of adult sociability, nociception, stereotypy, fear conditioning, and general anxiety. We then measured the effects on a composite measure of multivariate emotionality, derived from behavioral measures with high Cronbach's alpha coefficient of internal consistency. We then measured protein expression of various glutamate N-methyl-D-aspartate receptor subunits (GluN) and calcium/calmodulin-dependent protein kinase II (CaMKII) in the primary somatosensory cortex (S1), dorso-anterior hippocampus (dHip), ventro-posterior hippocampus (vHip), and amygdala (AMY), as well as induced corticosterone (CORT) in the plasma, dHip, vHip, and AMY. We went on to explore protein content correlates of behavioral emotionality, and if protein expression levels were modulated by prenatal and environmental conditions. Last, we used Agglomerative Hierarchical Cluster Analysis (HCA) to classify individual animals into subgroups, and profiled them to identify associations between prenatal exposure, environmental history, neurobiological profiles and a spectrum of behavioral outcomes. The results show that VPA rats reared in an enriched but predictable environment, do not develop autistic-like hyper-emotional behaviors, while those exposed to enriched but unpredictable environments or to standard environments develop a range of hyper-emotionality and other autistic symptoms, to an extent that depends on individual neurobiological correlates. This suggests that individuals exposed to an autism risk factor may become more neurobiologically sensitive to unpredictable environmental conditions than animals without risk factor exposure, and that rearing in a predictable, enriched environment could prevent the development of detrimental behaviors.

## Materials and methods

All procedures are detailed in the Supplementary Information Materials and Methods section. All animal experiments conformed to Swiss animal protection legislation.

### Animals and housing

Outbred Wistar Han male offspring were exposed prenatally on E11.5 (from vaginal plug on E0) to VPA (i.p. 500 mg/kg) or saline, as previously (Rodier et al., 1997; Favre et al., 2013), and reared from age P23 to P123 in 1 of 3 different postnatal home environments, with *ad libitum* chow and water. The *standard* laboratory housing environment (ST) housed 3 animals per cage, with bedding and cardboard tube, in a shared room, handled by various caretakers. The 2 enrichment environments were in an isolated housing room, handled by the same experimenters, where animals were housed in larger cages with ethologically positive multimodal stimuli (Smith and Corrow, 2005), including 6 animals per cage, running wheel, toys, treats and odors. The *unpredictable and enriching* environment (UE) was defined by twice weekly exchange in part of stimuli identities, totaling in 4 different settings (recycled). The *predictable and enriching* environment (PE) had a constant setting with the same set of enriching elements (no identity exchanges). Final sample size n_total_ = 107 males, 6 groups (SAL-ST, SAL-UE, SAL-PE, VPA-ST, VPA-UE, VPA-PE), *n* = 18 per group, except for n_VPA−PE_ = 17. Siblings were distributed for equivalent litter composition across environments (litters n_SAL_ = 12, n_VPA_ = 18).

### Behavioral and biochemical measures

We collected a battery of behavioral measures from age P81 to P123, in the order presented (conditions counterbalanced every 6 animals per group). We extracted plasma and fresh brains 10–13 min after the Elevated-Plus-Maze (EPM). We quantified plasma content of *corticosterone* (CORT) induced by EPM exposure with a competitive enzyme immunoassay (EIA, Assay Designs) following manufacturer's instructions. We macrodissected brain regions of interest (ROI) (Paxinos and Watson, 1998): *primary somatosensory cortex* (S1); *amygdalar complex* (Amy); *dorsal anterior hippocampus* (dHip), and *ventral posterior hippocampus* (vHip). Brain tissue was homogenized by sonication and total protein content quantified with bicinchoninic acid kit (Thermo Scientific - Pierce Biotechnology). Brain ROI samples were subject to typical gel electrophoresis Western Blot (WB) essay to measure total unbound peptide tissue content of: N-methyl-D-aspartate (NMDA) receptor subunits *GluN1* (116 kDa), *GluN2A* (180 kDa), and *GluN2B* (180 kDa), the secondary messenger calcium/calmodulin-dependent protein kinase two, *CaMKII* (50 kDa), and the glucocorticoid receptor, *GR* (90–95 kDa), as well as reference proteins *alpha-tubulin* (50–55 kDa), *beta-actin* (42–45 kDa), glyceraldehyde 3-phosphate, *GAPDH* (36 kDa). We obtained background subtracted immune-reactivity (IR) volumes with densiometric quantification (Bio-Rad) for each band. IR data from each band was normalized as a percent of the average IR of SAL-ST per essay (3 total consecutive essays, group counterbalanced); we used no prior normalization to in-lane reference proteins, because these were modulated by experimental group conditions (Figure S3).

## Results

### Social discrimination behavior

**Figure 1:**
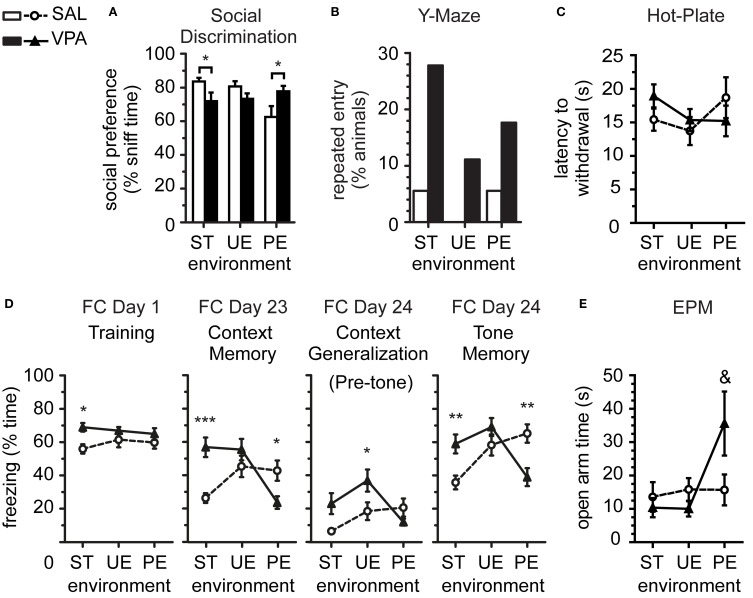
**Predictable enrichment effects on sociability, stereotypy and nociception, and reduction of fear and anxiety in VPA group. (A)** Preference for social stimulus sniffing over total stimuli sniffing time in Social Discrimination test was reduced in VPA vs. SAL in ST, and reversed in the VPA relative to SAL when in PE but not in UE environments. **(B)** Number of animals spontaneously re-entering the same arm in the Y-Maze was greater in VPA vs. SAL when pooled, but no significant effects when split by environment (shown here), where rare counts prevented clear interpretation. **(C)** Latency to hind paw withdrawal in the Hot-Plate test was variable, with no statistically significant effects. **(D)** Pavlovian Fear Conditioning (FC) showed that long-term enhanced fear (freezing) in VPA vs. SAL in ST was reduced specifically in PE, whereas both enrichment conditions enhanced memory in SAL. **(E)** Total time spent in the open arms of the elevated-plus maze (post-battery EPM), generally low in most groups, indicated reduced anxiety in some VPA animals reared in PE (*F*-test higher variance). Data in **(A,C–E)** show mean ± s.e.m. for each group. Sample sizes: SAL n_ST_ = 18, n_UE_ = 18, n_PE_ = 18; VPA n_ST_ = 18, n_UE_ = 18, n_PE_ = 17. Asterisks denote significant VPA × SAL mean comparisons, Bonferroni corrected, ^*^p < 0.05, ^**^p < 0.01, ^***^p < 0.001. Ampersand denotes significant VPA × SAL variance comparisons ^&^*p* < 0.01. VPA, valproic acid; SAL, saline; ST, standard environment; UE, unpredictably enriching environment; PE, predictably enriching environment.

We assessed sociability in the *Social discrimination test*, where animals were placed in a 3-chambered rectangular box, and given the choice (10 min) for voluntary sniffing between two novel stimuli located at opposite sides from the central chamber. The stimuli were: one social stimulus (a novel juvenile male rat) and one non-social stimulus (a novel inanimate object). We measured the time (s) spent exploring the different stimuli, and calculated a preference index for social sniffing over total time sniffing object and social stimuli. We observed first, a significant 2-ANOVA interaction between prenatal exposure and environment [Figure S1A; *F*_(2, 101)_ = 5.68, *p* = 0.005], in addition to main effects [prenatal *F*_(1, 101)_ = 3.97, *p* = 0.049; environment *F*_(2, 101)_ = 10.96, *p* < 0.0001], on total time sniffing novel stimuli (social + object); these effects were characterized *post-hoc* with Bonferroni corrected 2-sample comparisons, where we showed a reduced total sniffing time by VPA relative to SAL in the ST environment (*p* < 0.01), but not in UE (*p* > 0.5) or PE (*p* > 0.5). Further analysis revealed that such reduced total novelty exploration by VPA rats in the ST environment, compared to SAL animals, was due to a specific reduction in time exploring the social stimulus, as measured by reduced social preference index in VPA [Figure 1A; *T*-Test Welch's corrected ST *t*_(22)_ = 2.10, *p* = 0.048]. VPA and SAL animals reared in the UE showed no difference in social preference [*T*-Test UE *t*_(33)_ = 1.63, *p* = 0.116]. In the PE, however, VPA rats showed stronger social preference than SAL rats [*T*-Test PE *t*_(25)_ = 2.11, *p* = 0.046, Welch's corrected].

In summary, we observe the social withdrawal in VPA compared to SAL, when reared in ST, is prevented after rearing in a predictable enriched environment. In contrast, predictable environmental enrichment led to reduced novelty exploration and social preference in SAL group averages. We further observed that the variances of social preference index from VPA and SAL rats were significantly different in the ST and PE environments [*F*-test for variances ST *F*_(17, 17)_ = 5.57, *p* = 0.0009; PE *F*_(16, 17)_ = 4.14, *p* = 0.001], thus meeting the criterion for the application of Welch's correction on mean comparisons above, but not in the UE environment [*F*_(17, 17)_ = 1.13, *p* = 0.81], together suggesting that certain individuals may be more affected than others by different environmental conditions.

### Repetitive behavior

We measured stereotypy as the absence of spontaneous alternation between arms in 2 consecutive trials of the *Y-maze alternation test*. From the pooled data, we observed that a small number of animals (12 of 107 total sample) persevered by entering the same arm in consecutive trials, of which a significant majority were VPA-exposed rats (10 of 107, Fisher's exact test *p* = 0.015, 2-tailed). These results are consistent with enhanced stereotypy in VPA previously obtained in larger samples (Markram et al., 2008). However, separate analysis for smaller samples in ST, UE, and PE did not show significant differences between SAL and VPA animals, despite a trend for large effects (Figure 1B; Barnard's exact test ST *T* = 1.78, π = 0.05, *p* = 0.077; UE *T* = 1.45, π = 0.11, *p* = 0.146; PE *T* = 1.12, π = 0.71, *p* = 0.183).

### Nociceptive behavior

We assessed thermal nociception using the hot-plate test (Figure 1C), measured as the 1 trial latency (s) to hind paw withdrawal or jump from a heated (50°C) plate. There was no statistically significant 2-ANOVA interaction [*F*_(2, 101)_ = 1.43, *p* = 0.244], nor main effects [prenatal *F*_(1, 101)_ = 0.11, *p* = 0.745; environment *F*_(2, 101)_ = 0.95, *p* = 0.390] on latency to withdrawal, observed previously in bigger samples exposed to standard laboratory environments (Markram et al., 2008; Edalatmanesh et al., 2013).

### Pavlovian fear conditioning

We measured fear response as postural freezing in the *Pavlovian Fear Conditioning* (FC) paradigm, during 5 sessions: *Conditioning (Day 1)* in context-A, consisting of *Pre-Training* habituation (180 s) followed by a *Training* period (150 s) with 3 pairings between a tone (20 s) and an aversive foot-shock (1 s, 0.2 mA, 60 s inter-shock interval); *Context Memory Tests* (*Days 2* and *23*), in the conditioned context-A (480 s, no foot-shock or tone); *Tone Memory Sessions* (*Days 3* and *24*), consisting each of a *Pre-Tone* period in a novel context-B (180 s, without foot-shock nor tone), which was used as a form of context generalization test, followed by the *Tone Memory Test*, when the conditioned tone is continuously presented (330 s) in context-B, without foot-shock. We compared the effects of prenatal exposure and environment on group means with 2-ANOVA and *post-hoc* Bonferroni corrected 2-sample comparisons.

On *FC Day 1*, low freezing levels were observed overall during the *Pre-Training* period (Figure S1B), as expected, with no group effects [interaction *F*_(2, 101)_ = 1.53, *p* = 0.221; prenatal *F*_(1, 101)_ = 2.51, *p* = 0.116; environment *F*_(2, 101)_ = 0.98, *p* = 0.377]. During the *Training* period (Figure 1D), we observed a main effect of prenatal treatment [*F*_(1, 101)_ = 8.50, *p* = 0.004], with no other effects [environment *F*_(2, 101)_ = 0.19, *p* = 0.830; interaction *F*_(2, 101)_ = 0.91, *p* = 0.404]. *Post-hoc* analysis indicated higher freezing specifically by VPA compared to SAL in ST (*p* < 0.05), and no effect of VPA exposure in UE (*p* > 0.05) or PE (*p* > 0.05) environments.

During the *Context Memory* tests, contextually-cued freezing response in the short-term after training (*FC Day 2*, Figure S1B) showed a significant interaction [*F*_(2, 101)_ = 16.40, *p* < 0.0001], no main effect of prenatal exposure [*F*_(1, 101)_ = 0.17, *p* = 0.680], and a main effect of environment [*F*_(2, 101)_ = 3.20, *p* = 0.045]. *Post-hoc* tests revealed higher contextually-cued fear memory in the form of higher freezing by VPA than by SAL in the ST environment (*p* < 0.001), no difference in the UE environment (*p* > 0.05), and lower freezing by VPA than by SAL in the PE environment (*p* < 0.01). Similarly, the long-term *contextually-cued memory test* (*FC Day 23*, Figure 1D) showed a significant interaction [*F*_(2, 101)_ = 10.3, *p* < 0.0001], no main effect of prenatal exposure [*F*_(1, 101)_ = 2.73, *p* = 0.102], and a main effect of environment [*F*_(2, 101)_ = 5.11, *p* = 0.008], with *post-hoc* higher freezing by VPA than by SAL in ST (*p* < 0.001), no difference in UE (*p* > 0.05), and lower freezing by VPA than by SAL in PE (*p* < 0.05).

During the *Pre-tone* period, the short-term test (*FC Day 3*, Figure S1B) showed a significant interaction effect [*F*_(2, 101)_ = 12.09, *p* < 0.0001], without other effects [prenatal *F*_(1, 101)_ = 0.17, *p* = 0.680; environment *F*_(2, 101)_ = 1.31, *p* = 0.273], and *post-hoc* higher freezing to the non-conditioned context by VPA than by SAL in ST (*p* < 0.01), no difference in UE (*p* > 0.05), and lower freezing by VPA than by SAL in PE (*p* < 0.01). During long-term *Pre-tone* test (*FC Day 24*, Figure 1D), there was an interaction [*F*_(2, 101)_ = 4.52, *p* < 0.013], a main effect of prenatal exposure [*F*_(1, 101)_ = 4.57, *p* < 0.035], and a main effect of environment [*F*_(2, 101)_ = 4.03, *p* < 0.021], without significant *post-hoc* differences in ST (*p* > 0.05), but higher freezing by VPA than by SAL in UE (*p* < 0.05), and no differences in PE (*p* > 0.05).

During the *Tone* period, tone-cued freezing response in the short-term after training (*FC Day 3*, Figure S1B) showed an interaction [*F*_(2, 101)_ = 13.00, *p* < 0.0001], without other effects [prenatal *F*_(1, 101)_ = 0.48, *p* = 0.491; environment *F*_(2, 101)_ = 0.59, *p* = 0.557], and *post-hoc* higher freezing to the conditioned tone by VPA than by SAL in ST (*p* < 0.01), no difference in UE (*p* > 0.05), and lower freezing by VPA than by SAL in PE (*p* < 0.01). In the long-term tone-cued memory test (*FC Day 24*, Figure 1D), there was an interaction [*F*_(2, 101)_ = 11.00, *p* < 0.0001], no effect of prenatal exposure [*F*_(1, 101)_ = 0.34, *p* = 0.562], and a main effect of environment [*F*_(2, 101)_ = 4.80, *p* = 0.010], with *post-hoc* significant higher freezing by VPA than by SAL in ST (*p* < 0.01), no difference in UE (*p* > 0.05), but lower freezing by VPA than by SAL in PE (*p* < 0.01).

In summary, we consistently observed an interaction between prenatal exposure (SAL × VPA) and rearing environment (ST × UE × PE) on freezing levels, where the amplified fear memory in VPA reared in ST environment was prevented in rats reared in PE, but not in UE environments. In turn, VPA animals reared in UE environments presented freezing levels comparable to or higher than animals reared in ST environments. In contrast, SAL fear memory was enhanced by environmental enrichment (UE or PE), mostly irrespective of predictability, relative to the ST environment.

### General anxiety behavior

We assessed general anxiety behavior in the *Elevated-Plus Maze* test at the end of the behavioral test battery (post-battery EPM, 5 min.), where reduced exploration of the open arms indicated enhanced vigilance in novel, open spaces. Analysis of total distance moved during the test (Figure S1C) showed no 2-ANOVA interaction [*F*_(2, 101)_ = 1.52, *p* = 0.223] but a significant main effect of prenatal treatment [*F*_(1, 101)_ = 6.63, *p* = 0.012], a non-significant trend for a main effect of environment [*F*_(2, 101)_ = 2.93, *p* = 0.058]. *Post-hoc* comparisons revealed that VPA-exposed rats moved less than SAL rats in the ST (*p* < 0.05) but that there were no effects of VPA exposure in the PE (*p* > 0.05) or the UE (*p* > 0.05) environments. In addition, most animals showed high general anxiety, or vigilance, in the form of low (near 5%) exploration of the open arms, except for animals in the VPA-PE group. This is noted by higher variance in VPA group than SAL reared in the PE environment [Figure 1E; *F*-test for variances PE *F*_(16, 17)_ = 4.06, *p* = 0.006], but not in other environments [ST *F*_(17, 17)_ = 2.37, *p* = 0.084; UE *F*_(17, 17)_ = 2.22, *p* = 0.109], leading to a trend higher average time in the open arms compared to SAL [*T*-test Welch's corrected PE *t*_(23)_ = 1.87, *p* = 0.074], but not in other environments [*T*-test ST *t*_(34)_ = 0.61, *p* = 0.549; *T*-test UE *t*_(34)_ = 1.43, *p* = 0.163]. In agreement with open arm trends, head-dipping investigatory movements over the open arm edges presented a significant interaction [Figure S1D; *F*_(2, 98)_ = 4.51, *p* = 0.013], no main effects [prenatal *F*_(1, 98)_ = 0.33, *p* = 0.568; environment *F*_(2, 98)_ = 0.88, *p* = 0.419], and *post-hoc* higher exploration of VPA relative to SAL in the PE (*p* < 0.05) but not in ST (*p* > 0.05) or UE (*p* > 0.05) environments. Together this data indicate that environmental enrichment predictability reduces vigilance in a novel environment specifically in the VPA.

### Multivariate emotionality score

**Figure 2:**
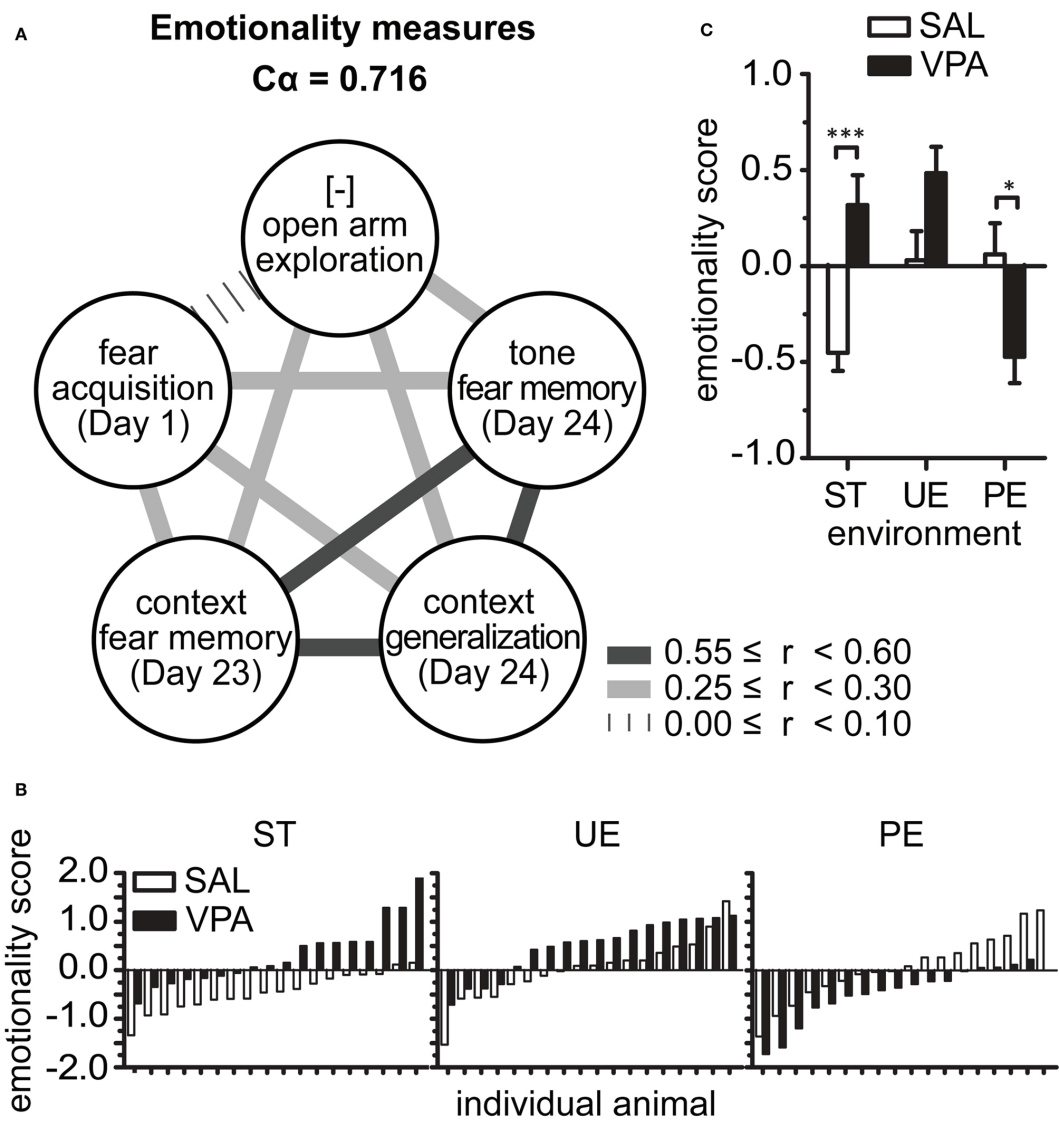
**Predictable enrichment reduces multivariate emotionality score in VPA group. (A)** Multivariate analysis identified 5 measures, representative of emotionality, with high (>0.7) Cronbach's alpha coefficient of internal consistency, which was computed from Pearson's correlation coefficients of z-transformed measures (mean *r* = 0.335). [-] denotes inverted scale, such that z-scores above zero represent high vigilance in EPM test and can be interpreted as increasing emotionality. **(B)** The emotionality score calculated as average of the 5 measures in **(A)**, per rat. **(C)** Group mean ± s.e.m. emotionality score indicated drastically opposite effects of environment on SAL and VPA: PE but not UE was required for VPA group to show reduced emotionality. Sample sizes: SAL n_ST_ = 18, n_UE_ = 18, n_PE_ = 18; VPA n_ST_ = 18, n_UE_ = 18, n_PE_ = 17. Asterisks denote significant VPA × SAL mean comparisons, Bonferroni corrected, ^*^p < 0.05, ^***^p < 0.001. VPA, valproic acid; SAL, saline; ST, standard environment; UE, unpredictably enriching environment; PE, predictably enriching environment.

We developed an *emotionality score* (modified from El-Kordi et al., 2013) based on the observation of high internal consistency between behavioral measures from the FC and EPM tests (Figure 2). Specifically, we found Cronbach's alpha coefficient of internal consistency (Cα) to be greatest when z-scores of 5 out of all behavioral measures were correlated (Figure 2A): *(1) Open arm exploration* (*EPM*, total time in open arm, inverted scale), *(2) Fear Acquisition* (*FC Day 1*, *Training* freezing % time), *(3) Long-term Context Fear Memory* (*FC Day 23*, freezing % time), *(4) Long-term Context Generalization* (*FC Day 24, Pre-tone* freezing % time), and (*5) Long-term Tone Fear Memory* (*FC Day 24, Tone* freezing % time). By inverting the scale of measure 1, we obtained *positive* bivariate linear correlations between each pair of variables (Figure 2A); the correlation coefficients between a given variable and all others were greater than 0.28, with a mean multivariate correlation of 0.335. These coefficients yield a high Cα of 0.716, indicating that these 5 measures have good internal consistency and can be combined to provide one composite score. Interestingly, these measures with high internal consistency represent various types of defensive response, be it in the face of danger (AFC Training), potential threat (EPM and FC Pre-tone), and learned threats (FC Context and Tone Memory). Thus, they capture various dimensions of an *emotionality* construct (Arnsten and Rubia, 2012; Fossati, 2012; Fernando et al., 2013). We averaged the 5 measures within an individual to obtain an *emotionality score* (Figure 2B), a multivariate composite indicator of emotionality from each animal, useful for evaluation of group effects and inter-individual differences.

Further analysis of the average emotionality scores per group showed that predictability is required for reduced emotionality in the VPA group (Figure 2C). In agreement with the univariate results for separate measures, the mean emotionality score per group showed a strong 2-ANOVA interaction [*F*_(2, 101)_ = 22.66, *p* < 0.0001], and main effects [prenatal *F*_(1, 101)_ = 5.52, *p* = 0.021; environment *F*_(2, 101)_ = 5.87, *p* = 0.004]. These effects were characterized *post-hoc* by higher emotionality by VPA vs. SAL reared in ST (*p* < 0.001), an effect entirely reversed in the predictable enriched environment, where VPA rats showed a significantly lower emotionality score than SAL rats in PE (*p* < 0.01). Importantly, no effect of VPA exposure was detected after UE (*p* > 0.05) environment.

These results show that even in an enriched environment, unpredictability still leads to hyper-emotionality in the VPA group. In parallel, we show that emotionality is enhanced by enrichment in SAL-exposed animals, but not to the level of those exposed to VPA.

### Correlation with protein expression

The behavioral results above demonstrated a general pattern of statistical interaction between prenatal and postnatal factors on behavior, where PE prevented autistic-like behaviors seen in VPA reared in non-predictable environments (ST and UE). In parallel, environmental enrichment in SAL, when compared to ST, favored enhanced fear memory and reduced social novelty exploration, and higher emotionality score, raising the question if autistic-like neurobiology was being induced in SAL after enrichment. We focused on the glutamatergic and corticosterone systems, both of which play an important role in emotion responses and cognition (De Kloet et al., 2009; Groeneweg et al., 2011; Sandi, 2011), are known to be affected by environmental enrichment (Sweatt, 2009; Pang and Hannan, 2013), and have been implicated in autism (Choudhury et al., 2012; Gaigg, 2012) and in the VPA model (Markram et al., 2007, 2008; Rinaldi et al., 2007; Silva et al., 2009). Bivariate Spearman's rho coefficients of correlation between emotionality score and protein expression indicate opposite patterns of correlation in SAL and VPA animals (Supplementary Information Results, Figure S2A). We also observed that grouping in the scatter plots (Figure S2B) suggested that environment was differentially implicated in the SAL and the VPA groups, a trend that was confirmed by significant univariate 1-ANOVA effects of environment on certain protein levels that were distinguishable between SAL and VPA groups (Figure S2C).

Together with behavioral results, the protein expression patterns indicated that enrichment did not induce VPA-like neurobiology in SAL. These results are in accordance with the literature in healthy and brain damaged animals, where enrichment improves learning, memory and stress reactivity functions (Supplementary Information Discussion). Importantly, these results suggest, indirectly, that VPA exposure modifies the neurobiological correlates of behavioral responsiveness to environmental predictability. Nonetheless, a direct association between prenatal VPA exposure, postnatal environment, neurobiology and behavioral outcomes required multivariate profiling of individuals, explored below.

### Inter-individual differences

We characterized individual differences with Hierarchical Cluster Analyses (HCA), done separately for SAL and VPA animals in order to identify multivariate characteristics that could distinguish animals exposed to an autism-risk factor. The analysis allowed profiling of subgroups of individuals based on a battery of behaviors, of relevance for the autism spectrum, and their association with environmental history and protein levels, in neural systems of emotion, cognition, and sensory function.

**Figure 3:**
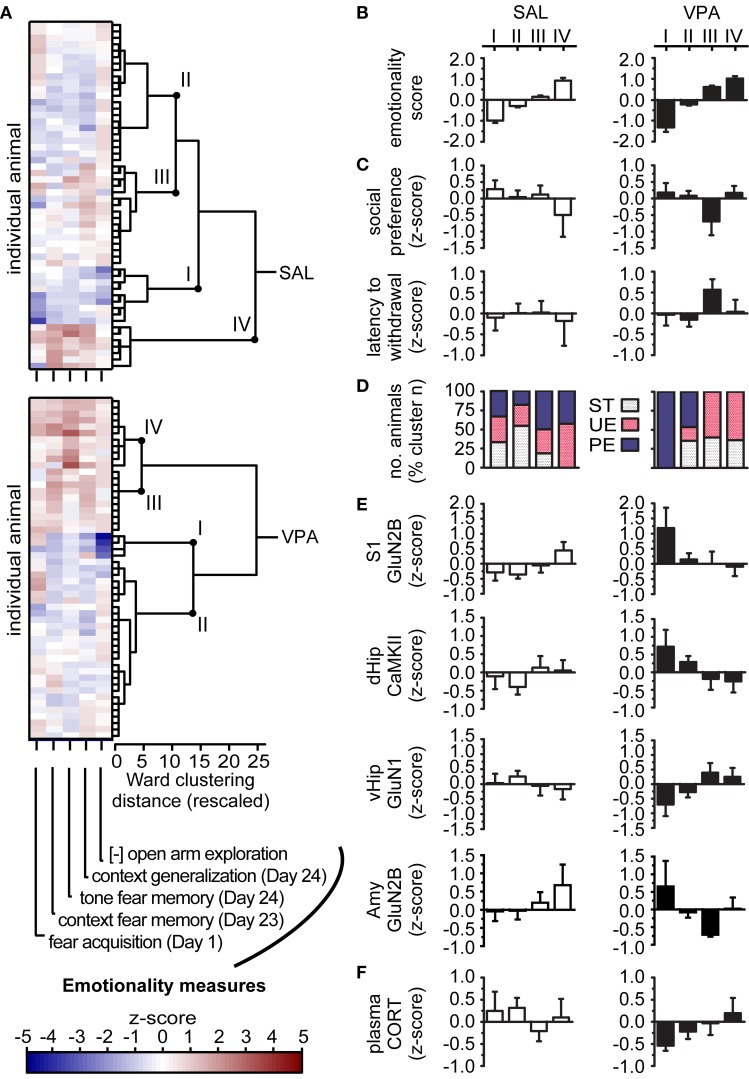
**Position on the autism-like behavioral spectrum depends on individual's VPA exposure, environmental history and neurochemical profile. (A)** Agglomerative Hierarchical Cluster Analysis (HCA) where we input z-scores (color bar) for each behavioral measure (horizontal-axis) from each rat (vertical-axis), and obtained dendrograms showing when individuals are joined into a cluster at increasing distances. [-] denotes inverted scale, such that z-scores above zero represent high vigilance in EPM test. Clusters identified as *I to IV* per group were selected to maximize size and within-cluster homogeneity, for the most parsimonious profiles. Sample sizes: SAL n_total_ = 54 animals, n_*I*_ = 17% of SAL animals, n_*II*_ = 41%, n_*III*_ = 29%, n_*IV*_ = 13%; VPA n_total_ = 53, n_*I*_ = 7%, n_*II*_ = 53%, n_*III*_ = 19%, n_*IV*_ = 21%. **(B)** Cluster core profiles, based on cluster average emotionality score, obtained after averaging input measures per individual rat. SAL and VPA exposed animals empirically segregated with increasing levels of emotionality score, but a greater proportion of VPA animals are hyper-emotional relative to SAL. **(C)** Cluster predicted profiles based on autism-relevant behaviors (social discrimination and hot-plate tests), not used as input in HCA; note only one VPA cluster has wide autistic-like behavioral spectrum. **(D)** Cluster predicted profiles based on environmental history, not used as input in HCA; note PE and non-predictable environments (ST or UE) segregated in VPA, but not in SAL. **(E,F)** Cluster predicted profiles, based on expression of Glutamatergic-related proteins in different brain regions and induced CORT in the blood (post EPM exposure), not used as input in HCA (SAL *n* = 51–54, VPA *n* = 50–53). Notice opposite protein expression patterns are associated with behavioral patterns and environmental history in VPA animals. Data in **(A–F)** presented as cluster mean ± s.e.m. VPA, valproic acid; SAL, saline; ST, standard environment; UE, unpredictably enriching environment; PE, predictably enriching environment. S1, primary somatosensory cortex; dHip, dorso-anterior hippocampus; vHip, ventro-posterior hippocampus; Amy, amygdala; GluN, glutamate N-methyl-D-aspartate receptor subunit; CaMKII, calcium/calmodulin-dependent protein kinase II; CORT, EPM-induced corticosterone.

To empirically determine an individual's membership to a given cluster, we input each animal's value from the 5 behavioral measures with large group effects demonstrated above, in AFC and EPM tests. We obtained one HCA dendrogram from the SAL and one from the VPA animals (Figure 3A), to represent the schedule when individual animals were joined into a cluster, at increasing distances in the multivariate space (Ward's Linkage of Squared Euclidean Distances). We evaluated the dendrogram for the most parsimonious cluster solution, seeking the largest possible clusters with high internal homogeneity, but with sufficient between-cluster separation to capture nuances in behavioral levels in different subgroups of animals. We identified four clusters from the SAL-exposed animals (SAL-*I* through SAL-*IV*) and four clusters from VPA-exposed animals (VPA-*I* through VPA-*IV*) with high internal homogeneity, as indicated by small within-cluster distances on the dendrogram, and with properties distinctive from other clusters, as indicated by larger between-cluster distances. Clusters sizes were: SAL n_total_ = 54, n_*I*_ = 9 (17%), n_*II*_ = 22 (41%), n_*III*_ = 16 (29%), n_*IV*_ = 7 (13%); VPA n_total_ = 53, n_*I*_ = 4 (7%), n_*II*_ = 28 (53%), n_*III*_ = 10 (19%), n_*IV*_ = 11 (21%). The strong difference in cluster sizes limited comparisons between clusters to qualitative, without statistical comparisons.

Given the input to determine cluster membership was the same set of variables integrated into the emotionality score above, HCA core cluster profiles could be obtained by first, averaging individual values into their emotionality scores, and then obtaining a cluster average emotionality score. Despite inter-individual differences in separate measures of emotionality (color range Figure 3A), and a wider range of scores observed in VPA relative to the range in SAL, the average emotionality score provided a single parsimonious descriptor of each clusters for discussion. We observed that VPA and SAL exposed animals both segregated in clusters with increasing mean emotionality levels (Figure 3B). We noted that a greater proportion of VPA animals (40%) are hyper-emotional, in clusters VPA-*III* and VPA-*IV*, relative to SAL (13%, in SAL-*IV*).

We proceeded to characterize attributes not used to determine cluster membership, with behaviors also relevant for the autism spectrum, but that showed group variance effects or small group mean effects, namely, social preference and Hot-Plate nociceptive threshold; Y-Maze stereotypy profiles were excluded because the counts were too low to draw meaningful conclusions. In the high emotionality clusters VPA-*III* and VPA-*IV*, only VPA-*III* also presented autistic-like wide spectrum of behavioral abnormalities, with the lowest levels of social preference, and the highest thresholds for thermal nociception (latency to withdrawal in the hot-plate test; Figure 3C). Importantly, the VPA subgroups with the lowest emotionality scores (VPA-*I*) or with intermediate emotionality scores (VPA-*II*) also displayed intermediate social preference and nociceptive thresholds. In the SAL group, however, there was only one high emotionality cluster (SAL-*IV*), with associated lower levels of social preference and, unlike VPA, low nociceptive threshold. Thus, none of the SAL clusters captured the full range of autistic-like behaviors displayed by VPA-*III*, indicating that control animals present an alternative relationship between social, sensory and emotionality behaviors than the relationship observed in animals exposed to VPA.

We evaluated if environmental history could explain such behavioral patterns (Figure 3D). Globally, there was a high degree of environmental history homogeneity within VPA clusters, where animals with a history of predictability often segregated from animals with a history lacking predictability (ST or UE). Specifically, 40% of VPA animals belonged to the high emotionality clusters (VPA-*III* and VPA-*IV*) and all had a history of environmental non-predictability (ST or UE environments). Interestingly, the range of behavioral effects of non-predictable environments in these clusters differed, where 19% of VPA animals (VPA-*III*) exhibited a wide range of autistic-like abnormalities, in emotionality, sociability and nociception, while 21% of VPA animals (VPA-*IV*) exhibited abnormalities circumscribed to hyper-emotionality. Importantly, we observed optimal outcomes in 7% of VPA animals (VPA-*I*), all reared in PE, indicating predictability is associated with the lowest emotionality scores in the study and no associated social or nociceptive abnormalities at the individual level. We also noted that 53% of VPA-exposed animals (VPA-*II*) did not show extreme behavioral levels on any measure, and were not characterized by any specific rearing environment. These results in VPA were in contrast to the patterns in SAL, where only in 13% of SAL animals (SAL-*IV*) had high emotionality scores, they were all reared in enriched environments, with no distinction between predictable and unpredictable environments. The majority of SAL-animals (70%, in SAL-*II* and SAL-*III*) presented intermediate behavioral scores that were not associated with any particular rearing environment. Together, these patterns indicate that predictability plays an exceptional role in determining behavioral outcomes in the VPA exposed individuals, compared to SAL controls. Nonetheless, the mixture of rearing environments in an 87% majority of SAL animals (SAL-*I*, SAL-*II*, SAL-*III*) and in a 53% small majority of VPA animals (VPA-*II*) indicate that, as expected in complex behaviors, prenatal and postnatal history alone are not sufficient to fully explain all behavioral outcomes. Interestingly also, within the VPA group itself we observe differential responsiveness to postnatal environment. Thus, further characterization of subgroups would require additional knowledge of the individual neurobiological factors that mediate the relationship between prenatal VPA exposure, environmental predictability and behavioral outcomes.

To identify candidate neurobiological mediators of these interactions, we evaluated cluster profiles of protein expression. We focused on the glutamatergic-related proteins (Figure 3E) and plasma corticosterone (Figure 3F), which showed univariate effects of VPA exposure or of environment above. We observed that the range and extent of the behaviors affected by environmental history depended on differential protein patterns between subgroups of individuals. We observed that VPA-*I*, the cluster with the lowest emotionality scores, after exposure to PE, also presented the highest levels of S1 Glu2B, dHip CaMKII, and Amy GluN2B, the lowest levels of vHip GluN1, and the lowest levels of plasma CORT. In contrast, VPA-*III* and VPA-*IV*, clusters with high emotionality scores, after exposure to non-predicable environments (ST and UE), presented the lowest levels of S1 GluN2B, dHip CaMKII and the highest level of vHip GluN1. Furthermore, we observed that these two hyper-emotional clusters segregated by the range of behaviors affected also had differential protein levels: VPA-*III* demonstrated the lowest level of Amy GluN2B; VPA-*IV*, on the other hand, presented the highest levels of plasma CORT. While these neurobiological differences remain associative, they suggest that individual differences in sensory and cognitive affective function are involved on how prenatal VPA and environmental predictability interact to determine a wide range of behaviors. Interestingly, the patterns observed in the SAL group were quite different: a given level of certain proteins observed in certain SAL and VPA subgroups (e.g., high levels of Amy GluN2B in SAL-*IV* and VPA-*I*), were associated with opposite behavioral levels between the subgroups. Additionally, SAL animals with high emotionality scores, low social preference and a wide range of low thermal analgesia, segregated after both types of enrichment, and thus, did not distinguish predictability as the factor determinant of behavior. Thus, the proportion of animals whose behavior is modulated by environmental predictability levels is higher in the VPA than in the SAL group.

Together, these results demonstrate that animals exposed to VPA show greater sensitivity than SAL to predictability levels in their environment, where individual differences in responsiveness to predictability depend on an interaction between environment and neurobiology in widespread systems for sensory, social and cognitive-affective function.

## Discussion

This study demonstrates that predictable environmental enrichment prevents the development of autistic-like hyper-emotional behaviors in rats exposed prenatally to VPA. We also report that inter-individual variability in cognitive, sensory and affective systems are associated with the enhanced sensitivity to environmental enrichment predictability in individuals exposed to an autism risk factor. To our knowledge, this is the first demonstration that prenatal, postnatal and neurobiological factors interact to determine differential autistic-like outcomes, on multivariate behaviors in an animal model of autism. Our results suggest that neural sensitivity is a hallmark of autism, and have immediate implications for research and therapy.

We find that rats prenatally exposed to VPA and reared in a standard laboratory housing environment, offering little stimulation to the animals, are characterized by behavioral hyper-emotionality, in agreement with results from previous studies (Markram et al., 2007; Gottfried et al., 2013; Roullet et al., 2013). Importantly, we further demonstrate that an enriched, yet *unpredictable* environment also induces hyper-emotionality in VPA. Crucially, this hyper-emotionality observed in a wide range of non-predictable environments (standard or unpredictably enriched) is largely prevented by raising VPA-exposed rats in an enriched, yet predictable, safe and non-surprising, environment. These prenatal and postnatal interaction effects were striking for group average univariate defensive behaviors in the fear associative learning, memory and generalization to non-conditioned contexts, and general anxiety, as well as for a multivariate emotionality score, composed of these behaviors. We also observed smaller but significant effects on group average sociability, and trend effects on stereotypy, but no effects on nociceptive thresholds. We interpret the absence of VPA effects in these tests previously shown to be abnormal in VPA to be due to insufficient power in the current sample to detect group effects, an idea supported by identification of VPA effects on subgroups identified based on individual differences discussed below. In parallel, the only other study demonstrating environmental enrichment improved social, nociceptive and emotion-related behaviors in VPA rats, did not examine the role of environmental predictability, nor did it provide a multivariate indicator of environmental effects (Schneider et al., 2006). Thus, our results significantly contribute to our understanding of environmental interventions on emotional and behavioral outcomes in an animal model of autism, by showing that it is not environmental stimulation *per se* that improves autistic-like symptoms, but that predictability in the environmental enrichment is the essential feature.

The individual-based cluster analysis and profiling presented suggest that individual outcomes are determined by the interaction between prenatal VPA exposure, postnatal environment, and neurobiology of sensory, cognitive and affective systems. Since autism is characterized by a spectrum of symptoms of differing severity (American Psychiatric Association, 2013; Argyropoulos et al., 2013) and differing responses to treatment (Bent and Hendren, 2010; Brentani et al., 2013), the individual differences recapitulated in our individual-based profiles provide valuable translational insights. Ellis, Boyce and colleagues have recently assembled an increasing body of evidence in favor of their Differential Susceptibility Model, according to which evolution has favored genetic variability between individuals in their biological sensitivity to environmental conditions. This would explain why similar environments can produce a range of mental health outcomes (Ellis and Boyce, 2011). They compare individuals with low biological sensitivity to environmental conditions to *dandelions*, which can flourish in a broad range of environments; individuals with high biological sensitivity to the environment are compared to *orchids*, which are uniquely specialized and require very precise conditions to flourish. As such, orchid individuals are subject to negative outcomes in stressful, unpredictable and negligent environments but may also have exceptionally positive outcomes, when reared in enriched, predictable, and supporting environments. Considering that numerous genetic and environmental factors have been implicated in autism, but no factor alone is causative in all cases, it is possible that the biological threshold for the development of autistic abnormalities is lowered by various combinations of genetic and epigenetic factors (Buxbaum, 2009; Abrahams and Geschwind, 2010; Markram and Markram, 2010). Careful interpretation of the rat subgroups identified here, suggests that individuals exposed to an autism risk factor, such as prenatal VPA, become more sensitive to the environment, thus making them more *Orchid*-like than non-exposed individuals, and develop a range of autism-like features if not reared in the optimal environment.

In this study, no single environmental condition is directly associated with emotionality level in controls; instead, one subgroup (SAL-*IV*) shows high emotionality levels after environmental enrichment, irrespective of predictability. In contrast, the VPA group displayed a direct association between environmental history and behavior, which was particularly marked when individual neurobiology was included in subgroup profiles. One subgroup (VPA-*III*) presented a peculiar neurobiological state (e.g., lowest levels of Amy GluN2B) and autistic-like, broad-spectrum of behavioral abnormalities (high emotionality, low sociability, high nociception threshold) after rearing in non-predictable environments (ST or UE). These can be considered as models of *orchids with diagnostic autism*: hyper-sensitive individuals that respond to most environmental conditions as if they were detrimental, leading to a wide range of debilitating behaviors. By contrast, VPA-*I* can be considered as a group of *orchids with optimal autism outcome;* hyper-sensitive individuals that display extremely low emotionality and normal range of social and nociceptive behaviors, and an opposite level of many neurobiological proteins (e.g., highest levels of Amy GluN2B, S1 GluN2B, and dHip CaMKII and the lowest levels of plasma CORT, and vHip GluN1), when reared in an enriched and necessarily predictable environment. Another subgroup (VPA-*IV*), represents *orchids with autistic traits:* individuals with an autism risk factor who nonetheless display a degree of dandelion-like resilience to non-predictable environments, with fewer behavioral abnormalities circumscribed to defensive behaviors, and displaying a different neurobiology from hyper-sensitive autistic-like animals. Finally, VPA-*II* can be considered to model *dandelions without autism*, the least sensitive individuals, characterized by average outcomes that are independent of environmental history, and no distinguishing neurobiology, at least in the systems investigated. These profiles support the hypothesis that individuals exposed to an autism-risk factor develop enhanced sensitivity to their environment, leading in some cases to hyper-sensitivity. The observation that a broad range of environments can lead to abnormal outcomes, when they lack predictability and even when they are enriched, suggests that it is critically important to tailor environmental interventions to individual sensitivity.

We show that prenatal VPA exposure and postnatal environment interact to modulate expression of glutamatergic and glucocorticoid-signaling proteins in cortical sensory (S1) and socio-cognitive-affective systems (Amygdala, Hippocampus), and that these factors together may predict individual variability in adult defensive, social and nociceptive behaviors. These results indicate candidate systems that could be implicated in individual differential response to treatment in autism. However, further studies are needed to clarify whether genetic, prenatal and postnatal factors directly determine protein expression or whether these are the result of recovery or compensatory mechanisms at different stages of development. Such a complex interaction between genetic, prenatal and postnatal mechanisms is possible. First, VPA acts epigenetically as a Class I histone deacetylase (HDAC) inhibitor, which can alter early developmental cascades (e.g., Pax-6, Kim et al., 2014) and ultimately affect multiple neural functions (Cotariu et al., 1990; Contestabile and Sintoni, 2013; Graff and Tsai, 2013). For instance, it has been shown that prenatal exposure to VPA enhances expression of molecules associated with experience-dependent plasticity, in the rat fetal brain (e.g., BDNF, Almeida et al., 2014), and in the pup (e.g., S1 GluN2A, GluN2B, and CaMKII, Markram et al., 2007) and that these changes are associated with accompanying electrophysiological hyper-functionality in S1, medial prefrontal cortex and Amygdala (Rinaldi et al., 2007, 2008a,b; Markram et al., 2008; Silva et al., 2009; Sui and Chen, 2012; Kim et al., 2013), and ultimately developmental recovery of these effects in the adult medial prefrontal cortex (Walcott et al., 2011; Martin and Manzoni, 2014), and adult hippocampal hyper-function (Edalatmanesh et al., 2013). Second, functional overlaps between such VPA-induced changes in the brain, and known genetic-autism factors occur (Rout and Clausen, 2009; Argyropoulos et al., 2013). Thirdly, these same systems have also been implicated in the effectiveness of environmental enrichment to improve cognition and emotion regulation (Cotariu et al., 1990; Van Praag et al., 2000; Hannan, 2014), through epigenetic modulation including the NMDA receptor-mediated pathways, activity-dependent CaMKII activation and enhanced expression of BDNF (Graff and Tsai, 2013). Thus, it is possible that VPA exposure in an individual with few genetic predisposing factors, could trigger a molecular syndrome similar to the syndrome triggered entirely by genetic factors in a different individual, leading, in both cases, to enhanced neurobiological sensitivity to environmental stimulation. However, we are only beginning to understand the effects triggered by VPA exposure at different developmental stages, the reasons why different systems are affected (e.g., Oguchi-Katayama et al., 2013; Roullet et al., 2013), and their role in autism pathology. In the future, functional studies of the overlap between genetic, epigenetic and prenatal-exposure risk factors (Argyropoulos et al., 2013), including but not limited to VPA exposure, could provide valuable insights into the neurobiology of environmental sensitivity in autism.

In conclusion, our results support the notion that typical (SAL) and autistic-like (VPA) rat brains are differentially sensitive to predictability in the environment, and that rearing in predictably enriched environments can directly reduce fear, vigilance, and emotional hyper-reactivity in autistic-like rats, factors which prevent individuals from fully benefiting from and contributing to their surroundings. The multivariate techniques used in this study make it possible to unravel previously overlooked effects of environmental predictability (e.g., Schneider et al., 2006), and to detect striking interactions between prenatal risk factors, postnatal environment, and neurochemical content. The results in control animals in contrast, are consistent with optimized cognitive-affective processing commonly observed in the rodent environmental enrichment literature (Supplementary Information Discussion). These results suggest the autistic brain is unusually sensitive to rearing environment and that, like an orchid, it requires specific conditions to flourish. These findings are consistent with the Intense World Theory of autism, which proposes that autistic brains process and store information excessively, within neuronal microcircuits, together suggesting that a predictable enriched environment could provide a safe haven of structural anchors in a world of sensory and emotional overflow (Markram and Markram, 2010; Lai et al., 2014). Conversely, environments lacking predictability favor the development of hyper-emotionality, and can in certain individuals, favor the development of a wide range of detrimental outcomes, including social withdrawal and sensory abnormalities. Predictability is a known important feature of autism treatment, but the research and diagnostic efforts to this date emphasize approaching the autistic child as hypo-functional and in need of stimulation, a perspective which could be detrimental to the therapeutic outcome. Thus, one of the main contributions of this work is the comparison between enriched environments with different levels of predictability, where we observe that unpredictability can be severely detrimental, even in the presence of enrichment. As such, this study supports a paradigm shift in the approach to autism as enhanced biological sensitivity to the environment.

Ultimately, our conclusions demand clinical verification in autistic children, in studies specifically designed to compare the impact of overstimulation in early stages of development, with the benefits of structured, familiar and predictable enriched environments (Markram and Markram, 2010). Our results were obtained in an animal model and after post-weaning, life-long exposure to a given environment, but future studies are necessary to determine the type, duration and intensity of enrichment predictability to be effective, while remaining viable, in the human setting. It seems reasonable to expect that exposing a child to an enriched and highly predictable environment, early in life, would in the least sensitive cases cause no harm, and in the most sensitive cases foster exceptional outcomes. If autistic children are indeed more neurobiologically sensitive to the environment than other children, then predicable environmental stimulation tailored to an individual's specific hyper-sensitivity could significantly improve their quality of life, by preventing or ameliorating the debilitating autistic symptoms of sensory overload and anxiety. Importantly, such constructive interactions with a safe and predictable world at key developmental sensitive periods could enhance coping and succeeding in subsequent less structured or unfamiliar contexts, and give place to the development of individual abilities in attention, memory, pattern recognition, and many others, including exceptional talents.

## Author contributions

MF, DL, DC, MC, HM, and KM designed research; MF, DL, JM, DC, and MC performed research; MF analyzed data; MF, HM, and KM wrote the paper.

### Conflict of interest statement

The authors declare that the research was conducted in the absence of any commercial or financial relationships that could be construed as a potential conflict of interest.
