# Supplementary material for: Predictable enriched environment prevents development of hyper-emotionality in the VPA rat model of autism
Source: Front Neurosci. 2015 Jun 2;9:127. doi: 10.3389/fnins.2015.00127 (PMC4452729; doi:10.3389/fnins.2015.00127)
Supplement: Supplementary file 1 [file SupportingInformation.DOCX]

Supporting Information (SI)

**Predictable enriched environment prevents development of hyper-emotionality in the VPA rat model of autism.**

Mônica Regina Favre, Deborah La Mendola, Julie Meystre, Dimitri Christodoulou, Melissa Cochrane, Henry Markram, Kamila Markram^*^

Laboratory of Neural Microcircuits, Brain Mind Institute, École Polytechnique Fédérale de Lausanne, Lausanne, Vaud, Switzerland

**^*^ Correspondence:**

Kamila Markram

École Polytechnique Fédérale de Lausanne,

BMI-SV-LNMC-Station 15

1015 Lausanne, VD, Switzerland

kamila.markram@epfl.ch

SI.1. Materials and methods

SI.1.1. Animals and prenatal VPA model of autism

Pregnant outbred Wistar Han females rats (n = 45, Janvier Laboratories, France) were purchased after vaginal plug detection at embryonic day 0 (E0). On E11.5 ([Rodier et al., 1997](#_ENREF_13);[Favre et al., 2013](#_ENREF_5)), randomly assigned dams received a single intraperitoneal (i.p.) injection of either sodium salt of valproic acid (NaVPA, Sigma-Aldrich, 500 mg/kg, 3.3 ml/kg, in 0.9 % saline 150 mg/ml, pH 7.2-7.3, filtered at 0.22µm), or saline as a vehicle control (SAL, 0.9 %, 3.3 ml/kg), and allowed to term and to rear their unculled litters. *Male* offspring (SAL or VPA) were assigned upon weaning on postnatal day 23 (P23) to one of 3 different post-natal home cage environments (ST, UE or PE, described below; n_weaned_ = 109, n_final_ = 107, with n = 18/group, except n_VPA-PE_ = 17). Siblings were distributed for equivalent litter composition across environments (litters n_SAL_ = 12, n_VPA_ = 18).

SI.1.2. Postnatal housing environments

Animals were housed continuously in the same home cages for 14 weeks (P23-P123), with *ad libitum* chow and water, in a 12-12h light-dark cycle from 7:30am, under controlled humidity, temperature, and radio background noise. All cages were cleaned the same day weekly, and animals were handled by the same experimenter. The *standard* laboratory housing (ST) housed 3 animals per cage (polycarbonate, Type 3, 425 x 265 x15 mm, Plexx, B.V., Elst, The Netherlands), with bedding and cardboard tube (10 cm diameter), in a shared housing room and cleaned by animal caretakers. In contrast, both enrichment groups were maintained in a restricted access and isolated room, handled by the same experimenters, and housed in larger home cages (2 polycarbonate cages, each 590 x 380 x 20 mm, with 100 mm raised wire lids, Type 4, Plexx, B.V., Elst, The Netherlands, connected by a homemade acrylic tube, 250mm long x 100 mm diameter) complemented with ethologically positive multimodal stimuli ([Smith and Corrow, 2005](#_ENREF_14)): social housing of 6 per cage, running wheel, texturized ramp, hiding tubes, toys, dried fruit or cereal, and odorized tissue paper. The *unpredictably enriching* environment (UE) was created with 4 different home cage settings, defined by exchange in identity of part of the stimuli, twice a week (setting exchanged 32 times, including 16 home cage cleanings, every 84 ± 4h). The *predictably and enriching* environment (PE) had a constant setting (no identity exchanges, 16 home cage cleanings, every 168 h).

SI.1.3. Behavioral measures

We collected a battery of behavioral and biochemical measures in the order described, during the light-phase (9h-17h00), on non-cleaning days, from all animals divided in 3 sets of 36 animals (counterbalanced 6 animals/group). Ages refer to set 1, with other sets following within a week.

*Social discrimination test.* We measured sociability in the social discrimination test at P81. The apparatus consisted of a dark grey, open-top rectangular box ([~90 cm X 35 cm, 40 cm walls, adapted from Crawley, 2007](#_ENREF_2)), equipped with 2 transparent perforated cylinders (12 cm diameter) for stimuli placement on opposite sides of the box, in a dimmed-lit room, cleaned with 5% ethanol. Animals were habituated (20-30 minutes) to the empty chamber on a day preceding the test. On the test day, we individually placed each animal from their home cage into the chamber for a 15 min. session. After adaptation (5min.), animals were given free choice (10 min.) to explore one social (a novel juvenile male rat) and one non-social (an inanimate novel object) stimuli. Time exploring each stimulus (cylinder voluntary sniffing) were blind scored from video records, and used to calculate: total exploration time of novel stimuli (social + object) and, preference index for social stimulus (social / (object + social) x 100 %).

*Y-maze alternation test.* We tested repetitive behavior in the spontaneous alternation version of the Y-maze ([Yadin et al., 1991](#_ENREF_15)) at P88. Rats were removed from their home cage and individually placed in the start arm of a Y- shaped maze (gray polyvinyl plastic, each arm 50 x 10 x 50 cm), and allowed to freely enter one of the 2 other arms (trial 1), as determined by the crossing of 4 paws into the arm. The rat was then picked up and immediately returned to the start arm and allowed again to freely enter one of the 2 arms (trial 2). Re-entering the same arm in both trials served as an indicator of repetitive behavior.

*Hot-plate nociception test.* We measured thermal nociception threshold in the hot-plate test ([Chapman et al., 1985](#_ENREF_1)) at P89. The apparatus (TSE, Bad Homburg, Germany) was cleaned with 5% ethanol, set to 50°C, and rats were removed from their home cage and individually placed on the plate surrounded by protective cylinder to prevent escape. Latency to hind paw withdrawal or jump was noted. No animal reached the cut-off time of 1 min.

*Fear conditioning test.* We measured Pavlovian fear conditioning (FC) in an automated chamber (30 x 20 x 21 cm, PanLab, Barcelona, Spain). Fear response was blind scored as the time spent in an immobile stereotypical posture (freezing). On the *conditioning session (day 1*, at P95*)*, animals were individually placed in the chamber for pre-training habituation (180 s) in context-A, followed by training (150 s) with 3 presentations of a tone (20 s, 800 Hz, 80 dB), each co-terminated with an aversive foot-shock (0.2 mA scrambled, 1 s, 60 s inter-shock interval). In the *context memory tests (days 2* and *23*, at P96 and P117, respectively), animals were re-exposed to the conditioned context A (480 s), in the absence of foot-shock or tone. In the *tone memory tests (days 3* and *24,* at P97 and P118, respectively*)*, animals were exposed to a *pre-tone* period in a novel context-B (180 s), without foot-shock nor tone, followed by continuous presentation of the conditioned tone (330 s). The context-A chamber had 20 steel rods (~1.5 cm) floor, black plastic sidewalls and transparent Plexiglas door and ceiling, cleaned with ethanol (5 %), under low house-lighting, and animals were transported in a clean plastic cage with bedding. The context-B chamber had a smooth grey plastic floor, perforated and angled metal sidewalls, absent ceiling, green lighting, cleaning with lavender-bleach (5 %), and transportation was done by a different experimenter in an empty covered cage.

*Elevated-plus maze test.* We measured post-battery general anxiety (vigilance in novel open spaces) at P123 in the elevated-plus maze (EPM). Animals were individually placed for 5 min. in the apparatus consisting of two arms enclosed by sidewalls (49 x 10 x 42 cm) arranged at right angles from two open arms (42 x 10 cm), connected at their center by a transition region of 10 cm^2^, all elevated 1 m from the ground. Time spent in each arm and total distance traveled was automatically determined by video tracking software (Ethovision-Noldus, The Netherlands), while head dips into open space were manually and blind scored from video records (excluding corrupted videos from 3 animals, 2 SAL-ST and 1 VPA-PE).

SI.1.4. Biochemical measures

*Tissue treatment.* Animals were sedated (isofluorane) 10-13 minutes after the EPM, and trunk blood was collected (7-12 ml, in chilled heparin 200 µl/sample, 5000 I.U./ml; Liquemin, Drossapharm AG, Basel, Switzerland), centrifuged (4 °C, 20 min., 4000 rpm), then the plasma stored in aprotinin (10 µl/ml, 1.0 µg/ml; Roche, Mannheim, Germany) at -80 °C. Fresh brains were extracted excluding the olfactory bulb and brainstem posterior to the cerebellum, weighted, then fast-frozen in isopentane (methyl-2-butane, Merck, Darmstadt, Germany) at approximately -20°C, and stored at -80 °C. Macrodissections of the brain regions of interest (ROI) were done on a cryostat (-20°C) based on gross coronal anatomical landmarks ([Paxinos and Watson, 1998](#_ENREF_11)), and stored at -80 °C, from the following ROIs: *primary somatosensory cortex* (S1, 1.60 to 0.20mm AP from Bregma); *amygdalar complex* (Amy, AP -1.80 to -3.8 mm); *dorsal anterior hippocampus* (dHip, AP -1.80 to -3.80 mm, including fimbria, dentate gyrus and CA1,2,3,) and *ventral posterior hippocampus* (vHip, AP -4.16 to -5.80 mm, including DG, CA1,2,3 ventral Amon’s horn landmarks). Brain tissue was homogenized by sonication (GE130 Ultrasonic Processor) in sodium dodecyl sulfate buffer (SDS, 1%, 100 µl), and stored at -80 °C. We quantified total protein content relative to a standard curve of bovine serum albumin (BSA) following manufacturer’s instructions for bicinchoninic acid kit (Thermo Scientific - Pierce Biotechnology, USA) and optical densiometric reading at 600nm (20-2000 up/ml working range; Biotrak II Plate Reader, GE Healthcare-Amersham Biosciences, Sweden). Quantified samples were diluted for equal total protein loading in Western Blot (1 ug/ul aliquote in distilled water, 1/3 Blue Loading Buffer and heated 95°C, 5 min., 600 rpm) and stored at -80 °C.

*Enzyme immuno-assay (EIA).* We quantified plasma content of *corticosterone* (CORT) induced by EPM exposure with a competitive enzyme immunoassay (EIA DetextX, Assay Designs, Inc., Ann Arbor, MI, USA; CORT sensitivity 18.6 pg/ml) following manufacturer’s instruction for microplate optical densiometric reading (Biotrak II Plate Reader 400-800 nm, GE Healthcare-Amersham Biosciences, Sweden). Briefly, samples were diluted in dissociation reagent and assay buffer, loaded onto a donkey anti-sheep IgG microtiter plate, followed by EIA-DetextX corticosterone conjugate and sheep polyclonal anti-CORT antibody, incubated (1h, ~200 rpm, room temperature, RT), washed, then added 3,3′,5,5′-Tetramethylbenzidine (TMB, 30 min., RT), followed by stop solution (hydrochloric acid, 1 M) to detected horseradish peroxidase (HRP) activity (yellow, 450 nm). CORT concentration inversely proportional to bound yellow was interpolated from the CORT standard curve and readings from 2 aliquots from each animal averaged.

*Western blot assay (WB).* Brain ROI samples quantified for total protein, were subject to typical WB essay. Samples were loaded aside a standard (Precision Plus Protein All Blue, Bio-Rad, cat. 161-0373) for gel electrophoresis (10 % acrylamide gel, 100 mV, 15 min., then 175 mV, 50 min.), in chilled running buffer (glycine, Tris, SDS, dH_2_O), then transferred (100 mV, transfer buffer, 60 min.) onto a nitrocellulose membrane (0.45 µm). We cut the membrane at desired molecular weights and blocked each with skimmed milk (5 %) in Tris buffered saline tween (TBS-T, 1 h), then incubated with a primary antibody against the protein of interest (POI, see below), washed (3 x 10 min., TBS-T), incubated with the secondary antibody (see below), and washed (3 x 10 min., TBS-T). Membrane HRP immuno-stained chemi-luminescence was revealed with SuperSignal West Dura Extended Duration Substrate (Pierce, Thermo Fisher Scientific, Switzerland), and immune-reactivity detected with the ChemiDoc XRS system (Bio-Rad Laboratories AG, Switzerland), simultaneously for the entire sample of a given ROI and POI, and adjusted against saturation. We obtained background subtracted volume intensities with densiometric quantification (Quantity One software, version 4.6.3, Bio-Rad). The traditional normalization to in-lane reference proteins was inadequate due to their modulation by experimental conditions (*Figure S3A-F*). Immuno-reactivity (IR) volume (intensity*mm2) from each band was normalized as a percent of the average IR volume of SAL-ST per essay (3 counterbalanced consecutive essays). Samples with band artifacts, or undetectable reference proteins were excluded from the analysis (typical image examples in (*Figure S3G*). In one WB, we loaded 15 ug of total protein per sample per ROI, and measured total unbound peptide tissue content of the N-methy-D-aspartate (NMDA) receptor subunits *GluN1* (116 kDa, Synaptic System, cat. 114001, 1:2500, primary incubation overnight, O/N, at 4 °C), and *GluN2B* (180 kDa, BD Transduction Lab, cat. 610417, 1:2500, O/N, 4 °C), the secondary messenger calcium/calmodulin-dependent protein kinase two, *CaMKII* (50 kDa, Upstate, cat. 05-532, 1:2500, O/N, 4 °C), and the reference protein glyceraldehyde 3-phosphate, *GAPDH* (36 kDa, Abcam, cat. ab8245, 1:20000, O/N, 4 °C), each probed with HRP conjugated anti-mouse secondary antibody (Calbio Chem cat. 401215, 1:3000, 1 h, RT). In another WB for the same samples, we loaded 25 ug of total protein per sample, and measured unbound peptide tissue content of the NMDA receptor subunit *GluN2A* (180 kDa, Upstate, cat. 07-632, 1:1000, O/N, 4 °C), and the glucocorticoid receptor, *GR* (90 to 95 kDa, Santa Cruz, cat. sc-1004, 1:1000, O/N, 4 °C), each probed with HRP conjugated anti-rabbit secondary antibody (Invitrogen, cat. G21234, O/N, 4 °C), as well as reference proteins *alpha-tubulin* (50 to 55 kDa, Sigma, cat. t5168, 1:50000, O/N, 4 °C), *beta-actin* (45 kDa, Chemicon, cat. mab1501, 1:10000, O/N, 4 °C), and *GAPDH* (as above), each probed with HRP conjugated anti-mouse secondary antibody (Calbio Chem cat. 401215, 1:3000, 1 h, RT).

SI.1.5. Statistical analyses

*Univariate and bivariate analyses.* Statistical evidence for a difference is considered at *p*-values < 0.05. For categorical variables (Y-maze), 2-sample comparisons (SAL x VPA) were done with Fisher's exact test in Prism software (v5.00 Win, GraphPad Software, San Diego, CA, USA), or Barnard’s Exact test (in case of rare event counts, software Matlab R2009a, The MathWorks Inc., USA), and inferred interaction effects indirectly. For continuous variables, we determined severe departures from normality, and homogeneity of variances with the F-test (in Prism). Most variables were Gaussian and homoscedastic, and were tested for an interaction between prenatal exposure (SAL x VPA) and postnatal environment (ST x UE x PE), and main effects, with 2 Factorial Analysis of Variance (2-ANOVA), followed by Bonferroni corrected 2-sample comparisons (SAL x VPA), separately for each environment (in Prism). Exceptionally for Gaussian and heteroscedastic measures (social preference, EPM open arm time), we used Welch’s corrected 2-sample t-tests (SAL x VPA, in Prism), and inferred interaction effects indirectly. To test the effects of environment on protein content for each prenatal treatment separately, we used 1-ANOVA on ranks, followed by Bonferroni corrected t-tests, or Welch’s corrected 1-ANOVA followed by Games-Howell corrected multiple pairwise comparisons (in SPSS). Finally, we explored bivariate correlations between emotionality score (bellow) and each protein with Spearman’s coefficient rho (in Matlab). Cases with any missing data were excluded for a variable pair (final n = 15 to 18 per group).

*Multivariate behavioral emotionality score.* We developed a composite score, for multivariate behavioral characterization of individuals and groups ([adapted from El-Kordi et al., 2013](#_ENREF_4)). We z-transformed each continuous measure (n_sample_ = 107, z-score = (value – mean_sample_) / S.D._sample_ ), then inverted the scale (negative values into positive) of some measures such that *input* *positive z-scores* represent low sociability, high analgesia, high vigilance (EPM), high fear conditioning (FC); conversely, *input negative z-scores* represent higher sociability, low analgesia, high exploration of novel spaces, low fear conditioning. We then obtained the Cronbach’s alpha coefficient of internal consistency (Cα) between different sets of variables (in SPSS), as an indicator of multivariate average Pearson’s correlations, noting it is proportional to the number of input measures. Measures weakly correlated (*r* < 2.8) to all others were exchanged until a high coefficient (0.7 < Cα < 1) was achieved. We present the 5 most internally consistent measures, identify them as components of emotionality, and average them to obtain a multivariate emotionality score for each animal.

*Multivariate cluster analysis.* We performed Agglomerative Hierarchical Cluster Analysis (HCA, in SPSS) of individual animals, separately for VPA and SAL, to characterize behavior heterogeneity and their associations with prenatal, postnatal and neurobiological factors. We input the z-score for 5 measures with high Cα separately from each animal, and merged animals on a dendrogram at increasing squared Euclidean distances based on Ward’s algorithm, aimed at minimizing the within-cluster variability (sum of squared errors). The most parsimonious clustering solution was chosen for discussion, based on examination of the dendrogram to favor the largest possible clusters (in number of animals), but which were highly internally homogeneous (short within-cluster distances). Clusters were profiled in terms of their emotionality composite scores, calculated from variables input into the HCA and thus used for empirical cluster separation, and on non-input environmental history, behavioral and, neurobiological measures, used as predicted associated attributes of each cluster.

SI.2. Results

SI.2.1. Protein Expression

Given the general pattern of interaction between prenatal and postnatal factors on behavior, we investigated if the neurobiological correlates were also distinguishable between VPA and SAL, with separate analyses *(Figure S2)*.

Bivariate Spearman’s rho correlation coefficients indicated that SAL and VPA presented an overall pattern of opposite direction of associations between emotionality score and protein content (color code in *Figure S2A*). Certain molecules in the glutamate signaling system reached statistical significant monotonic correlation with emotionality score, including: *1) S1 GluN2B*, which was correlated with SAL emotionality, but show a trend anti-correlation with VPA emotionality; *2) dHip CaMKII*, which was correlated with SAL emotionality, but anti-correlated with VPA emotionality; *3) vHip GluN1*, which was anti-correlated with SAL emotionality, but correlated with VPA emotionality. The scatter plots of molecules selected to represent each system *(Figure S2B)* qualitatively indicate that postnatal environment could be differentially implicated in the association between neurobiology and behavior depending on prenatal VPA exposure. Importantly, molecules such as Amy GluN2B and plasma CORT presented a scatter with certain extreme values or non-monotonic in nature, which were not detected as significant in the Spearman’s analysis, but where environment may still have a noteworthy effect for some individuals.

As suggested by grouping in the scatter plots, some of the molecules associated with different behavioral levels in SAL and VPA were indeed differentially modulated by the environment in SAL and VPA groups *(Figure S2C)*. Globally, predictability affected VPA, while enrichment, in particular with unpredictability affected SAL. This was supported by a statistically significant effect of environment in VPA *S1 GluN2B* (*F*_2,50_ = 4.39, *p* = 0.017), characterized post hoc by higher VPA *S1 GluN2B* content after PE relative to UE (*p* = 0.017), without other statistically significant effects (ST x UE *p* = 1.000, ST x CE *p* = 0.137). We also observed an effect of environment in SAL *S1GluN2B* (*F*_2,32.2_ = 14.78, *p* < 0.0001, Welch’s corrected), characterized by higher S1 *GluN2B* content in UE relative to ST (*p* < 0.0001), without other statistically significant effects (ST x PE *p* = 0.280, UE x PE *p* = 0.070). We further observed an effect of environment in VPA *dHip CaMKII* content (*F*_2,31.5_ = 12.10, *p* < 0.0001, Welch’s corrected), characterized by significantly higher VPA *dHip CaMKII* in PE relative to ST (*p* < 0.0001) as well as in PE relative to UE (*p* = 0.044), but not in ST relative to UE (*p* = 0.457). We also observed an effect of environment in SAL *dHip CaMKII* content (*F*_2,27.9_ = 52.68, *p* < 0.0001, Welch’s corrected), characterized by significantly higher SAL *dHip CaMKII* in UE relative to ST (*p* < 0.0001) as well as in PE relative to ST (*p* < 0.0001), but not in UE relative to PE (*p* = 0.298). Similarly, we observed an effect of environment in VPA *Amy GluN2B* content (*F*_2,49_ = 4.45, *p* = 0.017), characterized post hoc by significantly higher in PE after UE (*p* = 0.022), without other statistically significant effects (ST x UE *p* = 1.000, ST x PE *p* = 0.137). We also observed an effect of environment in SAL *Amy GluN2B* content (*F*_2,30.0_ = 4.37, *p* = 0.022, Welch’s corrected), characterized by higher SAL *Amy GluN2B* in UE relative to ST (*p* = 0.031), without other statistically significant effects (ST x PE *p* = 1.000, UE x PE *p* = 0.096). Lastly, plasma CORT showed no effect of environment in either VPA (*F*_2,50_ = 1.20, *p* = 0.311) nor SAL (*F*_2,51_ = 0.51, *p* = 0.606). Together, these explorative patterns indicate that the neurochemical correlates of high emotionality in SAL are different to those in VPA, and are for some proteins and individual subgroups, paralleled by differential responsiveness to predictability in the enriched environment.

SI.3. Discussion

One issue to further discuss is the effect of enrichment in SAL rats. Despite autistic-like enhanced fear and reduced social preference reported here for SAL after enrichment, our data does not support our enrichment is inducing autistic-like features in SAL. Instead, we believe these VPA-ST and SAL-enriched behaviors are seemingly common endpoints of completely different neurobiological trajectories: one driven by VPA triggered autistic-like neuropathology, the other by environmentally enhanced neural function. The effects of enrichment in SAL should thus be accompanied by cognitive and adaptive enhancement not extensively measured here. Despite this limitation, the effects of enrichment on SAL behavior also agree with the literature in rodents, where enrichment was shown to improve cognition and emotion regulation ([Lewis, 2004](#_ENREF_9);[Fox et al., 2006](#_ENREF_6);[Pang and Hannan, 2013](#_ENREF_10);[Ravenelle et al., 2014](#_ENREF_12)). Specifically, while inconsistency in social isolation and group housing conditions limit direct contrast with our sociability measured here ([Graff and Tsai, 2013](#_ENREF_7);[Pang and Hannan, 2013](#_ENREF_10)), previous reports also demonstrated improved adaption in the form of reduced activity in novel contexts, comparable to our results in the social discrimination and EPM tests, in addition to enhanced cognitive function in contextual learning in the Morris Water maze, associative learning in FC, also seen here ([Diamond et al., 2007](#_ENREF_3);[Pang and Hannan, 2013](#_ENREF_10)). Furthermore, if our enrichment were inherently detrimental, than UE should have the highest effects, since unpredictability is a greater stressor ([Koolhaas et al., 2011](#_ENREF_8)), while we observe SAL-UE on average at the level of PE or between ST and PE for all behaviors. Alternatively, if our largest SAL effects, observed in PE, were due to a most detrimental environment, than the same environment should not recover anxiety and fear in already emotional VPA animals. Perhaps most importantly, we report opposite correlation patterns between neurobiological proteins and emotionality score, in SAL and VPA, and differential association with environmental history, which indicate that despite apparently similar behavioral levels between SAL-enriched and VPA-ST, their brains are not in the same state. Thus, other cognitive and operant tests, and functional molecular studies are needed to clarify the scope of environmental benefits to cognitive-affective functions. We cannot exclude the possibility that other SAL clusters with greater environmental homogeneity would be formed with other behaviors not measured here, or that enrichment aspects other than predictability might be of greater importance in distinguishing behaviors in SAL. Nonetheless, the contrast between SAL and VPA exposed animals clearly distinguish the association between an autism-risk factor and neurobiological sensitivity to the environmental predictability.

SI. References

Chapman, C.R., Casey, K.L., Dubner, R., Foley, K.M., Gracely, R.H., and Reading, A.E. (1985). Pain measurement: an overview. *Pain* 22, 1-31. doi: 10.1016/0304-3959(85)90145-9.

Crawley, J.N. (2007). Mouse behavioral assays relevant to the symptoms of autism. *Brain Pathol* 17, 448-459. doi: 10.1111/j.1750-3639.2007.00096.x.

Diamond, D.M., Campbell, A.M., Park, C.R., Halonen, J., and Zoladz, P.R. (2007). The temporal dynamics model of emotional memory processing: a synthesis on the neurobiological basis of stress-induced amnesia, flashbulb and traumatic memories, and the Yerkes-Dodson law. *Neural Plast* 2007, 60803. doi: 10.1155/2007/60803.

El-Kordi, A., Winkler, D., Hammerschmidt, K., Kastner, A., Krueger, D., Ronnenberg, A., Ritter, C., Jatho, J., Radyushkin, K., Bourgeron, T., Fischer, J., Brose, N., and Ehrenreich, H. (2013). Development of an autism severity score for mice using Nlgn4 null mutants as a construct-valid model of heritable monogenic autism. *Behav Brain Res* 251, 41-49. doi: 10.1016/j.bbr.2012.11.016.

Favre, M.R., Barkat, T.R., Lamendola, D., Khazen, G., Markram, H., and Markram, K. (2013). General developmental health in the VPA-rat model of autism. *Front Behav Neurosci* 7, 88. doi: 10.3389/fnbeh.2013.00088.

Fox, C., Merali, Z., and Harrison, C. (2006). Therapeutic and protective effect of environmental enrichment against psychogenic and neurogenic stress. *Behav Brain Res* 175, 1-8. doi: 10.1016/j.bbr.2006.08.016.

Graff, J., and Tsai, L.H. (2013). Histone acetylation: molecular mnemonics on the chromatin. *Nat Rev Neurosci* 14, 97-111. doi: 10.1038/nrn3427.

Koolhaas, J.M., Bartolomucci, A., Buwalda, B., De Boer, S.F., Flugge, G., Korte, S.M., Meerlo, P., Murison, R., Olivier, B., Palanza, P., Richter-Levin, G., Sgoifo, A., Steimer, T., Stiedl, O., Van Dijk, G., Wohr, M., and Fuchs, E. (2011). Stress revisited: a critical evaluation of the stress concept. *Neurosci Biobehav Rev* 35, 1291-1301. doi: 10.1016/j.neubiorev.2011.02.003.

Lewis, M.H. (2004). Environmental complexity and central nervous system development and function. *Ment Retard Dev Disabil Res Rev* 10, 91-95. doi: 10.1002/mrdd.20017.

Pang, T.Y., and Hannan, A.J. (2013). Enhancement of cognitive function in models of brain disease through environmental enrichment and physical activity. *Neuropharmacology* 64, 515-528. doi: 10.1016/j.neuropharm.2012.06.029.

Paxinos, G., and Watson, C. (1998). *The rat brain in stereotaxic coordinates.* San Diego: Academic Press.

Ravenelle, R., Santolucito, H.B., Byrnes, E.M., Byrnes, J.J., and Donaldson, S.T. (2014). Housing environment modulates physiological and behavioral responses to anxiogenic stimuli in trait anxiety male rats. *Neuroscience* 5, 76-87. doi: 10.1016/j.neuroscience.2014.03.060.

Rodier, P.M., Ingram, J.L., Tisdale, B., and Croog, V.J. (1997). Linking etiologies in humans and animal models: studies of autism. *Reprod Toxicol* 11, 417-422. doi: 10.1016/S0890-6238(97)80001-U.

Smith, A.L., and Corrow, D.J. (2005). Modifications to husbandry and housing conditions of laboratory rodents for improved well-being. *ILAR J* 46, 140-147. doi: 10.1093/ilar.46.2.140.

Yadin, E., Friedman, E., and Bridger, W.H. (1991). Spontaneous alternation behavior: an animal model for obsessive-compulsive disorder? *Pharmacol Biochem Behav* 40, 311-315. doi: 10.1016/0091-3057(91)90559-K.

SI Figure legends

Figure S1: Prenatal exposure and postnatal environment interact for effects on various behavioral measures. (A) Total sniff time of novel stimuli, either social (juvenile rat) or object, in Social Discrimination test, indicate decreased novelty exploration in VPA relative to SAL at ST, where environmental enrichment (UE and PE) decreases it in SAL-ST. (B) Fear Conditioning (FC) shows that short-term (see main text for long-term) enhanced fear in VPA versus SAL in ST is reduced specifically in PE, whereas both enrichment conditions enhance context memory in SAL, and only PE has the greatest enhancement of context generalization and tone memory in SAL. *Pre-training (*FC Day 1) shows expected low total freezing in the chamber prior to tone-shock conditioning; *Context memory test* (FC Day 2), total freezing during conditioned context-A presentation, without tone and foot-shock; *Context generalization (*FC Day 3), total freezing during pre-tone period in a non-conditioned context-B, without foot-shock; *Tone memory test* (FC Day 3) total freezing during conditioned tone presentation, in a non-conditioned context-B, without foot-shock. (C) Total distance moved in the elevated-plus maze (EPM post-battery), and (D) Number of head dipping exploratory movements into the open spaces in the EPM, supports trends of reduced vigilance specifically in VPA-exposed animals as observed in open arm time trends. See main text for statistical results. Notes: Data show mean ± s.e.m for each group. Sample sizes: SAL n_ST_ = 15-18, n_UE_ = 15-18, n_PE_ = 15-18; VPA n_ST_ = 15-18, n_UE_ = 15-18, n_PE_ = 17, all males. Asterisks denote significant VPA x SAL comparisons, * *p* < 0.05, ** *p* < 0.01, *** *p* < 0.001. VPA, valproic acid; SAL, saline; ST standard environment; UE unpredictably enriching environment; PE predictably enriching environment.

**Figure S2: Neurobiological correlates of emotionality are opposite between VPA and SAL groups, and are specifically modulated by predictability in VPA group. (A)** Correlogram for each prenatal group representing bivariate rho coefficient (colorbar; ^#^ denotes smallest *p*-values, 0.01 ≤ *p* ≤ 0.05) between emotionality scores and different protein levels in different brain regions and in the blood plasma; Western Blot measures are normalized, as % SAL-ST; EIA measures of CORT shown as ng protein of interest / ug of total protein). The direction of the relationships reveals opposite molecular levels in the SAL and VPA brains correlate with a given behavioral levels, in particular for S1 GluN2B, S1 CaMKII, dHip CaMKII, vHip GluN1. Sample sizes: SAL n = 51 to 54, VPA n = 50 to 53. **(B)** Scatter plot matrix (z-scores from individual rats) for selected proteins in each region of interest and emotionality score, to illustrate relationship patterns. Note animals with similar emotionality levels in SAL and VPA groups do not present the same protein levels and postnatal environment groups tend to pool differentially. **(C)** Univariate effects of postnatal environment on proteins levels selected as in B. Note expression of some proteins are modulated by environment with a particular effect of PE in VPA not seen in SAL. **Notes:** Data in C show mean ± s.e.m for ranks of protein levels used in A and B. Asterisks denote significant ST x UE x PE comparisons,* *p* < 0.05. Sample sizes: SAL n_ST_ = 15-18, n_UE_ = 16-18, n_PE_ = 15-18; VPA n_ST_ = 17-18, n_UE_ = 18, n_PE_ = 16-17. VPA, valproic acid; SAL, saline; ST standard environment; UE unpredictably enriching environment; PE predictably enriching environment; S1, primary somatosensory cortex; dHip, dorso-anterior hippocampus; vHip, ventro-posterior hippocampus; Amy, amygdala; GluN, glutamate N-methyl-D-aspartate receptor subunit; CaMKII, calcium/calmodulin-dependent protein kinase II; CORT, induced corticosterone.

**Figure S3: Typical 'housekeeping' proteins unexpectedly modulated by experimental conditions. (A-D)** GAPDH immunoreactivity (IR) is modulated by prenatal exposure to VPA and/or by postnatal housing environment, to different extents depending on brain region: **(A)**  in S1, effect of environment (*F*_2,99_ = 4.58, *p* = 0.013), with higher levels in UE than in ST or in PE; **(B)** in dHip, trend interaction (*F*_2,98_ = 2.48, *p* = 0.089) and with SAL-PE higher than other groups and significant higher than VPA-PE (*p* = 0.008), **(C)** in Amy, effect of environment (*F*_2.101_ = 6.42, *p* =0.002), with UE lower than in ST or UE. **(D)** in vHip, effect of environment (*F*_2,97_ = 4.52, *p* = 0.013) and of VPA-exposure (*F*_1,97_ = 5.20, *p* = 0.025), with lower levels in UE than in ST or UE in particular for VPA-exposed animals. **(E-F)** alongside D, demonstrate modulation by prenatal VPA exposure and/or by postnatal environment in the same essay in the same brain region (vHip) depend on the protein: **(E)** on alpha-tubulin, effect of VPA exposure in ST with increased in VPA-ST relative to SAL-ST (*t*_21.4_ = 3.88, *p* = 0.0008, Welch's corrected); **(F)** on actin, no significant effects, thus differ from same-essay proteins in D and E. Thus, these proteins typically considered to have stable expression are in fact modulated by our experimental conditions and thus were deemed inadequate Western Blot in-lane loading controls, and thus not used for normalization of IR from other proteins of interest (POI), discussed in main text. POI's are instead normalized as percent of same-essay SAT-ST average IR. See main text for statistical results. Data in A-F show mean ± s.e.m for each group. A-F Sample sizes: SAL n_ST_ = 15-18, n_UE_ = 15-18, n_PE_ = 15-18; VPA n_ST_ = 15-18, n_UE_ = 15-18, n_PE_ = 17. **(G)** Typical Western Blots, from the 1^st^ set of 36 animals (out of 3 sets), each set with 6 animals per group and counterbalanced for essay conditions. Horizontal black line separates different essay conditions, where white horizontal lines limit where the membrane was cut differently, and vertical white lines indicate separate gels. Animals identified with columns numbers 14 and 34 were excluded from analysis because there were no detectable reference proteins. **Notes:** Asterisks denote significant VPA x SAL comparisons, ** *p* < 0.01, *** *p* < 0.001. VPA, valproic acid; SAL, saline; ST standard environment; UE unpredictably enriching environment; PE predictably enriching environment.
