# Supplementary figures and images for: Predictable enriched environment prevents development of hyper-emotionality in the VPA rat model of autism
Source: Front Neurosci. 2015 Jun 2;9:127. doi: 10.3389/fnins.2015.00127 (PMC4452729; doi:10.3389/fnins.2015.00127)

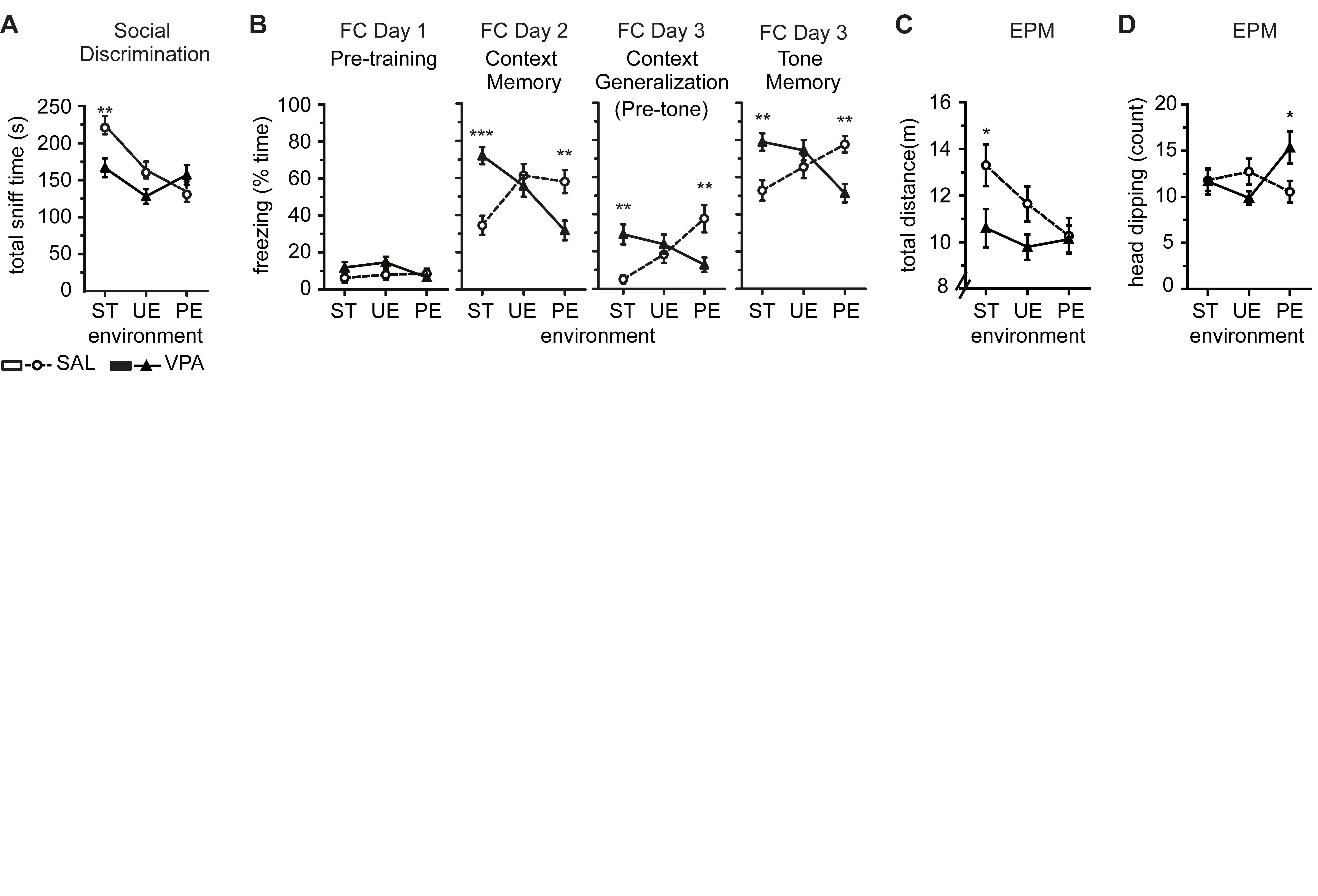

Supplement: Supplementary file 2 [file Image1.TIF]
